# Editorial: Understanding molecular mechanisms to facilitate the development of biomarkers for therapeutic intervention in gastrointestinal diseases and sepsis

**DOI:** 10.3389/fgene.2025.1581299

**Published:** 2025-03-11

**Authors:** Dipak Kumar Sahoo, Romy M. Heilmann, Ashish Patel

**Affiliations:** ^1^ Department of Veterinary Clinical Sciences, College of Veterinary Medicine, Iowa State University, Ames, IA, United States; ^2^ Department for Small Animals, Veterinary Teaching Hospital, College of Veterinary Medicine, University of Leipzig, Leipzig, Saxony, Germany; ^3^ Department of Life Sciences, Hemchandracharya North Gujarat University, Patan, Gujarat, India

**Keywords:** gastrointestinal disorders, inflammatory bowel diseases, ulcerative colitis, molecular biomarkers, sepsis, colorectal cancer, disease diagnosis

**FIGURE 1:**
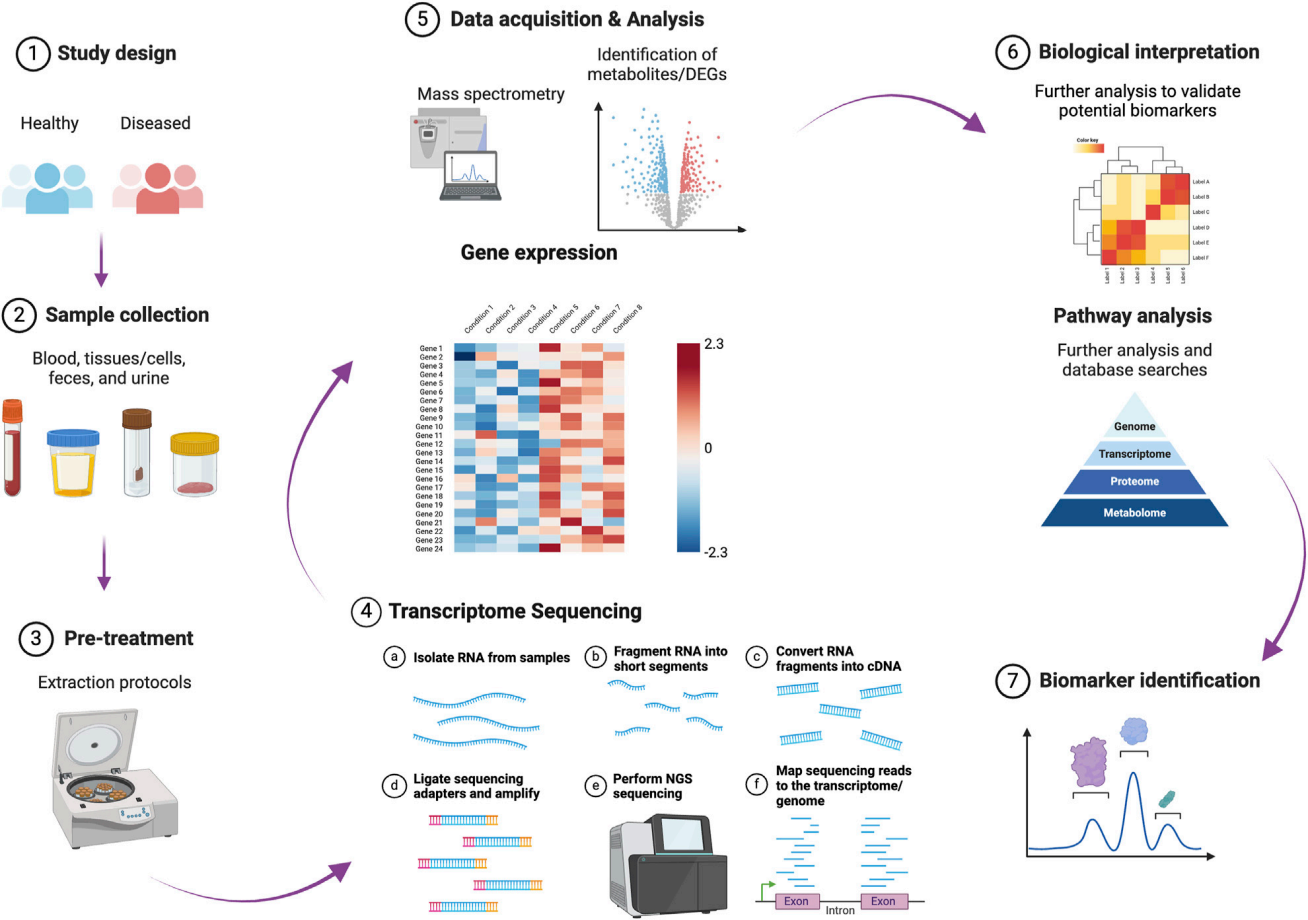
A schematic representation of the different phases involved in identifying and validating molecular biomarkers for disease diagnosis and prognosis. This figure was created in BioRender (https://BioRender.com/d22b503). DEGs: differentially expressed genes.

Gastrointestinal (GI) disorders include a range of pathological conditions with varying severities and outcomes that impact the integrity and function of the GI tract. These conditions include indigestion, the inflammatory bowel diseases (IBDs) ulcerative colitis (UC) and Crohn’s disease (CD), and malignant tumors. The resulting dysfunction of the intestinal barrier, leading to impaired permeability, allows for the translocation of luminal contents, including intact microbes and microbial products. This situation can cause severe sepsis and potentially fatal outcomes if timely intervention is not provided. Advancements in omics technology have facilitated the identification and evaluation of molecular biomarkers for disease diagnosis. These encompass genomic (e.g., single nucleotide polymorphisms), transcriptomic (including non-coding RNAs), epigenetic (e.g., DNA methylation), proteomic, metabolomic, and microbiome biomarkers (Dalal et al., 2020; Sahoo et al., 2024a; Sahoo et al., 2022b; Figure 1). These biomarkers hold significant clinical potential for improving diagnosis, prognosis, and treatment strategies in patients with GI disorders and sepsis.

For diagnosing sepsis, in addition to lactate as a widely utilized biomarker, other surrogate markers, such as C-reactive protein (CRP) and procalcitonin, which are produced in response to infection and inflammation, may assist in identifying patients at risk of developing severe sepsis before significant organ dysfunction occurs (Faix, 2013). As oxidative stress (OS) plays a key role in the progression of sepsis and septic shock to multiple organ failure (Sahoo et al., 2024b; Wong et al., 2025), OS markers associated with sepsis, specifically, superoxide dismutase, soluble endoglin, asymmetric dimethylarginine, and neopterin, merit further clinical investigation (Helan et al., 2022). Several emerging biomarkers such as microRNA-486-5p, circular RNAs (circRNAs), HOXA distal transcript antisense RNA (a lncRNA located on chromosome 7q15.2), protein C (a vitamin K-dependent glycoprotein), prokineticin 2, and triiodothyronine also hold potential for enhancing early detection and prognostic assessment of sepsis with high sensitivity and specificity (He et al., 2024).

Mendelian randomization (MR) examines the causal effects of modifiable exposures, such as potential risk factors, on health by utilizing genetic variants linked to those specific exposures (Richmond and Smith, 2022). MR analysis by Zhang et al. showed that CMPF (3-carboxy-4-methyl-5-propyl-2-furanpropanoate) has an association with 28-day all-cause mortality in clinical cases of sepsis. The metabolic pathway of alpha-linolenic acid and linoleic acid was identified as a crucial factor in the development and progression of sepsis. The study by Ye et al. demonstrated that levels of soluble suppression of tumorigenicity 2 (sST2) in the blood have significant clinical diagnostic and prognostic implications in sepsis. Moreover, sST2 showed a comparable predictive capability to the SOFA (Sequential Organ Failure Assessment) and APACHE II (Acute Physiology and Chronic Health Evaluation II) scores and had a greater predictive capability than lactic acid levels in assessing the prognosis of patients with sepsis. Jin et al. used single-cell RNA sequencing (scRNA-seq) and identified several diagnostic markers for sepsis, such as PIM1 (proviral integration site for Moloney murine leukemia virus kinase 1), HIST1H1C (Histone Cluster 1 H1 Family Member C), and IGSF6 (Immunoglobulin Superfamily Member 6). The involvement of PIM1 in modulating the immune-inflammatory response during sepsis was verified through experimental validation, indicating that PIM1 is a promising novel therapeutic target.

The endoscopic evaluation of patients suspected of IBD with the collection of mucosal biopsies for histopathological confirmation continues to be the gold standard for establishing an IBD diagnosis, assessing treatment efficacy, and identifying post-operative recurrence; however, it is associated with high costs and invasiveness. Biomarkers enable non-invasive disease evaluation, with C-reactive protein and fecal calprotectin being the most frequently utilized biomarkers in current clinical practice. The T>C substitution SNPs that affect the functionality of the *DLG5* (discs large homolog 5) protein, along with the lack of *CARD15*/*NOD2* (caspase recruitment domain family number 15/nucleotide-binding oligomerization domain-containing protein 2) SNPs associated with CD pathogenesis, could serve as genomic biomarkers (Dudzińska et al., 2018). Several potential microbiome biomarkers (microbial markers) have been reported. For example, *Faecalibacterium prausnitzii* and its phylogroups and elevated *Escherichia coli* counts serve as potential biomarkers for CD diagnosis. *Akkermansia muciniphila* is identified for pediatric CD diagnosis. Additionally, reductions in *Firmicutes* (*Clostridiales*) levels correlate with IBD severity, while increased abundance of *Lactobacilllaceae* and *Enterococcaceae* families, as well as the genera *Lactobacillus*, *Enterococcus*, and *Eggerthella*, are noted in UC patients (Wang et al., 2024). In dogs with CIE, there is a notable dysbiotic profile in both luminal and mucosal intestinal bacteria, marked by a reduction in *Clostridium* and *Bacteroides* and an increase in *Enterobacteriaceae* (Sahoo et al., 2022a).

The regulation of gene expression mediated by microRNA (miRNA) is essential for the appropriate development and functioning of the intestine (Shanahan et al., 2021). Numerous studies have effectively identified unique miRNA profiles that indicate the upregulation or downregulation of one or more miRNAs in intestinal biopsy samples from patients with IBD (James, 2020) and dogs affected with chronic inflammatory enteropathy (CIE) (Sahoo et al., 2024a). The colonic mucosa of patients with active UC was shown to overexpress specific miRNAs, including miR-21, miR-150, and miR-155, and have a reduction in miRNAs like miR-143 and miR-145 when compared to healthy controls (Alghoul et al., 2022). Comparing the colonic mucosa of patients with active CD and healthy controls, there was an upregulation of miR-196 and a downregulation of miR-7 (Alghoul et al., 2022). The differential expression of miRNAs in saliva, blood, and colon tissue samples was analyzed in UC and CD patients (Schaefer et al., 2015). This research highlighted multiple miRNAs (specifically, miR-21, miR-31, miR-142-3p, miR-142-5p) with expression levels exhibiting significant changes across all three sample types when comparing IBD patients to non-IBD controls (Schaefer et al., 2015). Recent studies indicate that DNA methylation of specific genes contributes to the pathogenesis of IBD, implying their potential utility as clinical biomarkers (Cooke et al., 2012; Nimmo et al., 2012). A comprehensive analysis of methylation patterns across the genome, performed on rectal mucosal biopsies, revealed specific differential gene signatures, including Fanconi anemia complementation group (FANCC), thyroid hormone receptor-associated protein 2 (THRAP2), globoside alpha-1,3-N-acetylgalactosaminyltransferase 1 (GBGT1), tumor necrosis factor ligand superfamily member 4 (TNFSF4), TNF superfamily member 12 (TNFSF12), docking protein 2 (DOK2), and fucosyltransferase 7 (FUT7). These genes exhibited notable differences in methylation levels in specimens from patients with CD or UC compared to healthy individuals (Cooke et al., 2012). Response to infliximab treatment in patients with IBD resulted in notable decreases in macrophage-derived cluster of differentiation 14 (CD14) and CD86 levels, along with the chemokine CCL2 (Magnusson et al., 2015), highlighting their potential as surrogate biomarkers to monitor IBD patients during treatment.

The research by Song et al. employing MR suggests that interleukin-13 (IL-13) contributes to the pathophysiology of IBD (CD and UC). Whereas macrophage migration inhibitory factor appears to be specifically related to CD, stem cell factor is more likely to play a role in the progression of IBD (CD and UC). Another MR study by Qian et al. showed that patients with gastric cancer exhibit decreased blood levels of tryptophan, nonadecanoate (19:0), and erythritol. Zhu et al. employed MR and demonstrated that the genes *GPBAR1* (G protein-coupled bile acid receptor 1), *IL1RL1* (Interleukin 1 receptor-like 1), *PRKCB* (Protein Kinase C Beta), and *PNMT* (Phenylethanolamine N-Methyltransferase) are linked to an increased risk of IBD. Whereas *IL1RL1* was shown to have a protective effect against the risk of CD, *GPX1* (Glutathione peroxidase 1), *GPBAR1*, and *PNMT* are implicated in the risk of UC. In a scRNA-seq study by Keever-Keigher et al., common expression patterns were observed in GI disorders, including an extensive upregulation of MTRNR2L8 (MT-RNR2 Like 8) across various cell types. The increase of XIST (X Inactive Specific Transcript) expression across different cell types in individuals with UC and an elevated expression of Th2 (T helper 2)-associated genes in eosinophilic disorders is also noteworthy.

Colorectal cancer (CRC) is a common GI neoplasia. To facilitate the early detection of CRC, a minimally invasive and reproducible technique known as liquid biopsy (LB) has been established. This method isolates cancer-derived components from the patients’ peripheral blood and/or other body fluids, including circulating tumor cells (CTC), miRNA, long non-coding RNAs (lncRNAs), and circulating tumor DNA (ctDNA) (Zhang et al., 2023). Heat-shock protein 27 (Hsp27) has been identified as expressed explicitly in well-differentiated CRC and is linked to other significant CRC biomarkers, such as epidermal growth factor receptor (EGFR), tumor necrosis factors, protein kinase B (AKT), and human epidermal growth factor receptor 2 (ERBB2) (Gan et al., 2014). Glutathione S-transferase pi1 (GSTP1) and KTR8 were overexpressed in both well-differentiated and poorly differentiated CRCs. In contrast, triosephosphate isomerase (TPI), tubulin beta chain (TUBB), and fatty acid-binding protein (FABP1) were upregulated exclusively in well-differentiated CRCs. Human leukocyte antigen A (HLA-A) was observed to be increased in poorly differentiated CRC (Gan et al., 2014). Circular RNAs (circRNAs) such as hsa_circ_001978, hsa_circ_103627, hsa_circ_105039, and circ_0124554 may also serve as indicators for a diagnosis of CRC. Levels of serum miR-21, miR-29a, and miR-125b can potentially differentiate patients with early colorectal neoplasia from healthy ones (Yamada et al., 2015). Serum miRNAs, such as miR-21, miR-92a, miR-182S, and miR-223, along with other miRNAs like miR-17–5p, miR-18a–5p, miR-18b–5p, miR-103a–3p, miR-127–3p, miR-151a–5p, and miR-181a–5p may have clinical utility as biomarkers in the non-invasive screening for CRC (Zhang et al., 2023). CircRNAs have the potential to synergize with various proteins or RNAs, demonstrated by circ_0000523 and methyltransferase-like 3 (METTL3), to enhance the accuracy of a CRC diagnosis (Wang et al., 2022).

Carcinoembryonic antigen (CEA) and carbohydrate antigen 19-9 (CA19-9) currently serve as the primary serum tumor markers for assessing the prognosis of CRC. The research conducted by Dai et al. indicates that both the overall survival rate and the disease-free survival rate in patients with CRC progressively decline with an increasing number of positive tumor markers before and after surgical intervention. Nomograms utilizing pre-and postoperative CEA and CA19-9 demonstrate high accuracy in predicting survival and recurrence for stage I-III CRC patients following radical surgery, significantly outperforming the American Joint Committee on Cancer (AJCC) 8th Tumor-Node-Metastasis (TNM) stage. The research by Rad et al. highlights the significant impact of machine learning algorithms on predicting CRC recurrence, specifically by examining the least number of serial CEA measurements necessary for accurate prediction of recurrence. The research conducted by Pan et al. involved a retrospective analysis of clinical data from 36,708 patients who underwent gastroscopy and colonoscopy between 2005 and 2022. Conventional adenomas (CAs), serrated polyps (SPs), non-adenomatous polyps (NAPs), and CRC were all associated with an increased risk of *Helicobacter pylori* infection and older age. The presence of moderate to severe intestinal metaplasia was associated with an increased risk of NAP and CAs. The risk of CRC was found to be increased with low-grade intraepithelial neoplasia, whereas gastric cancer was linked to high-grade intraepithelial neoplasia. A correlation was also observed between advanced gastric pathology and an increased risk of CRC.

In individual patients without clinical signs, biomarkers or biomarker panels have the potential to serve as a significant resource for screening to identify cancer at an early stage or recognize precancerous conditions. For symptomatic patients, these biomarkers can help differentiate cancerous from benign states. Furthermore, in cancer patients undergoing treatment such as surgical procedures, radiation therapy, and/or chemotherapy, surrogate disease biomarkers are valuable tools for evaluating the success of tumor elimination (complete resection and remission) and the potential for disease recurrence. Identifying and validating optimal biomarkers and biomarker panels is a crucial step, as it offers considerable potential for enhancing personalized medicine and overall clinical outcomes. However, further research into the specificity of molecular biomarkers for sepsis, IBD, and CRC is necessary before they can be utilized as diagnostic tools in clinical practice.
